# Facilitators of and barriers to reducing thirty-day readmissions and improving patient-reported outcomes after surgical aortic valve replacement: a process evaluation of the AVRre trial

**DOI:** 10.1186/s12913-020-05125-5

**Published:** 2020-03-27

**Authors:** Stein Ove Danielsen, Philip Moons, Marit Leegaard, Svein Solheim, Theis Tønnessen, Irene Lie

**Affiliations:** 1 Center for Patient-centered Heart and Lung Research, Department of Cardiothoracic Surgery, Division of Cardiovascular and Pulmonary Diseases, Oslo University Hospital, Building 63, Ullevål, PO Box 4956, Nydalen, 0424 Oslo, Norway; 2 Institute of Clinical Medicine, Faculty of Medicine, University of Oslo, Oslo, Norway; 3 KU Leuven Department of Public Health and Primary Care, KU Leuven-University of Leuven, Leuven, Belgium; 4 Department of Nursing and Health Promotion, Faculty of Health Sciences, Oslo Metropolitan University, Oslo, Norway; 5 Institute of Health and Care Sciences, University of Gothenburg, Gothenburg, Sweden; 6 Department of Paediatrics and Child Health, University of Cape Town, Cape Town, South Africa; 7 Center for Clinical Heart Research, Department of Cardiology, Division of Medicine, Oslo University Hospital, Ullevål, Oslo, Norway; 8 Department of Cardiothoracic Surgery, Division of Cardiovascular and Pulmonary Diseases, Oslo University Hospital, Ullevål, Oslo, Norway

**Keywords:** Surgical aortic valve replacement, 30-day readmission, Post-discharge telephone intervention, Process evaluation, Implementation

## Abstract

**Background:**

The Aortic Valve Replacement Readmission (AVRre) randomized control trial tested whether a telephone intervention would reduce hospital readmissions following surgical aortic valve replacement (SAVR). The telephone support provided 30 days of continuous phone-support (hotline) and two scheduled phone-calls from the hospital after discharge. The intervention had no effect on reducing 30-day all-cause readmission rate (30-DACR) but did reduce participants’ anxiety compared to a control group receiving usual care. Depression and participant-reported health state were unaffected by the intervention. To better understand these outcomes, we conducted a process evaluation of the AVRre trial to gain insight into the (1) the dose and fidelity of the intervention, (2) mechanism of impacts, and (3) contextual factors that may have influenced the outcomes.

**Methods:**

The process evaluation was informed by the Medical Research Council framework, a widely used set of guidelines for evaluating complex interventions. A mix of quantitative (questionnaire and journal records) and qualitative data (field notes, memos, registration forms, questionnaire) was prospectively collected, and retrospective interviews were conducted. We performed descriptive analyses of the quantitative data. Content analyses, assisted by NVivo, were performed to evaluate qualitative data.

**Results:**

The nurses who were serving the 24/7 hotline intervention desired to receive more preparation before intervention implementation. SAVR patient participants were highly satisfied with the telephone intervention (58%), felt safe (86%), and trusted having the option of calling in for support (91%). The support for the telephone hotline staff was perceived as a facilitator of the intervention implementation. Content analyses revealed themes: “gap in the care continuum,” “need for individualized care,” and “need for easy access to health information” after SAVR. Differences in local hospital discharge management practices influenced the 30-DACR incidence.

**Conclusions:**

The prospective follow-up of the hotline service during the trial facilitated implementation of the intervention, contributing to high participant satisfaction and likely reduced their anxiety after SAVR. Perceived less-than-optimal preparations for the hotline could be a barrier to AVRre trial implementation. Integrating user experiences into a mixed-methods evaluation of clinical trials is important for broadening understanding of trial outcomes, the mechanism of impact, and contextual factors that influence clinical trials.

**Trial registration:**

ClinicalTrials.gov, NCT02522663. Registered on 11 August 2015.

## Background

Severe aortic stenosis (AS) is the most common reason for surgical aortic valve replacement (SAVR) [1]. Calcification of the heart valve leaflets is a prominent cause of altered cardiac blood flow, a pathology that leads to AS [1]. Non-rheumatic aortic stenosis, or valve degeneration associated with aging, requires intervention, leading to more invasive surgeries like SAVR and less-invasive ones like transcatheter aortic valve implantation (TAVI). The frequency of these two treatments is accelerating in Western countries because of growing aging populations [2]. The prevalence of AS in the US has risen from less than 1% for people < 44 years old to 13.3% for people > 75 years old [3]. For patients > 65 years, long-term mortality after SAVR surgery is very low, with a median survival of 11–13 years [4, 5]. However, hospital readmissions after SAVR are common. A recent meta-analysis of 30-day all-cause readmission (30-DACR) rates after SAVR showed an average rate of 17%, with substantial variability across countries and different assessment methods [6].

Hospital readmissions have a high societal and economic burden and significant impact on healthcare systems. Readmissions during the rehabilitation phase also affect the quality of life of patients and their caregivers [7, 8]. Frequent causes for readmissions after SAVR are atrial fibrillation, infections, and heart failure [6, 9]. For these reasons and the institutional and personal burdens mentioned, healthcare providers aim to reduce readmissions. Also, governments enact legislation that provides incentives to reduce hospital readmissions [10]. Research results on the efficacy of interventions aimed at preventing readmissions is ambiguous to date [11–14]. Hence, more rigorous research is needed that uses robust experimental designs. One such study is the Aortic Valve Replacement Readmission (AVRre) trial [15]. This intervention involved the following.

From August 2015 to April 2017, we conducted the AVRre trial [15]. This randomized controlled trial (RCT) tested the effectiveness of a post-SAVR discharge telephone service from which participants could get healthcare information. It tested specifically whether a 24/7 patient-activated telephone hotline and intermittent, scheduled telephone follow-ups (TFUs) would reduce the 30-DACR rate, alleviate symptoms of anxiety and depression, and improve patients’ self-perceived health state. A control group received usual post-discharge care [15]. The AVRre RCT was ineffective in reducing the 30-DACR rate [16]. However, the 24/7 hotline and TFU were effective in reducing symptoms of anxiety during the first month after surgery. Symptoms of depression and the patients’ self-perceived health state remained unchanged after SAVR surgery for up to 1 year [16].

In order to understand why the intervention failed in some aspects and succeeded in others, we conducted a process evaluation that was informed by the Medical Research Council (MRC) guidelines [17]. Our process evaluation aimed to gain insight into (1) the appropriateness of the AVRre intervention dose (i.e., number of days and calls administered) and fidelity of the intervention (i.e., delivered as designed); (2) the mechanism of positive/negative impacts; and (3) the contextual factors that may have influenced the intervention in unanticipated ways. The aim of the present study was to report the process evaluation of the AVRre trial.

## Methods

### Overview of completed AVRre intervention

To better understand our process evaluation goals and results, we first describe the original AVRre RCT. Before implementing the AVRre trial, we developed an evidence-based manual for use with a 24/7-hotline telephone service and completed a pilot study of its use. To refine the intervention, we considered users’ experiences obtained from research interviews with former cardiac surgery patients (Supplemental file 3). The hotline staff nurses and the project coordinator were educated on the purpose of the intervention and were trained to administer it effectively. During the AVRre trial, the hotline was staffed by experienced nurses [15]. The nurses were closely assisted and monitored through consultations with the project coordinator. These consultations included regular case discussions. Nurses also participated in educational sessions with expert physicians and a physiotherapist.

The intervention group was offered a 24/7 (around-the-clock) telephone service, and patients received a structured telephone follow-up that was conducted by the project coordinator on days 2 and 9 after discharge. The patients’ answers from the two follow-ups were recorded in a form and later analyzed. Patients also completed questionnaires 1, 3, 6, and 12 months after surgery [15].

### Framework for and design of process evaluation of AVRre trial

While outcome evaluations assess the effectiveness of an intervention in producing change (in this case, reduction in 30-DACR), process evaluations help researchers see *how* an intervention outcome or impact was achieved and if it was implemented as intended [17]. To carry out the process evaluation of the AVRre trial, we used the updated MRC framework for developing and evaluating complex interventions [18]. This version guided the process evaluation of relevant aspects of the AVRre trial. Ideally, a process evaluation should start at the feasibility and pilot phases of a proposed intervention and be followed by periodic prospective evaluation during the AVRre trial implementation phase [17]. Done in parallel with the outcome evaluation, the process evaluation provides additional important insight about whether the intervention activities of the RCT were implemented as intended and why or why not they were effective.

We used quantitative and qualitative methods to carry out the process evaluation. Mixed-methods research is rigorous and uses multiple types of data to leverage the strengths and offset the weaknesses of each data type. This approach aids in real-life contextual understanding of a clinical intervention from multi-level perspectives [19]. Our MRC-informed process evaluation first evaluated aspects of the intervention implementation. We assessed design and implementation aspects prior to and during the AVRre trial. We aimed to determine whether preparation and execution of the hotline manual was adequate, and whether the pilot study and education and training program influenced the fidelity of intervention delivery. We also aimed to determine whether the dose (i.e., number of days and calls administered) of the intervention was given as planned, and whether the follow-up calls done by the project coordinator influenced the fidelity. Secondly, we sought to determine the mechanism of impact by analyzing AVRre trial participants’ responses to the intervention using several data sources, like field notes, questionnaires, and the nurses’ feedback from individual consultations, team case discussions/consultations, and a focus group interview of the nurse staff experiences. Lastly, we analyzed patient-reported data (questionnaire and narratives) and medical records’ data to local hospitals’ discharge patterns of the SAVR patients, for example, readmission length of stay. Table 1 gives the overview of the process evaluation of the AVRre trial hotline intervention.

**Table 1:** Overview of the process evaluation of the AVRre trial

Process elements	Scope of the process evaluation	Data type	Data sources	Analysis methods
Implementation	a) What was the process like in delivering the intervention? b) How was the delivery of the intervention conducted in terms of dose^b^ and fidelity^c^?	Qualitative and quantitative Qualitative and quantitative	Field notes and memos, mind maps^a^, registration forms, questionnaires, journal records, and focus group interviews	NVivo and content analysis, qualitative assessments, and descriptive analyses
Mechanisms of impact	How did the patients react to the intervention?	Qualitative and quantitative	Field notes and memos, questionnaires, registration forms, observations, and a focus group interview	NVivo and content analysis, qualitative assessments, and descriptive analyses.
Context	Were there any contextual factors^d^ that might have substantially influenced the intervention and the primary outcomes?	Qualitative and quantitative.	Field notes and memos, questionnaires, and a focus group interview	NVivo and content analysis, and descriptive analyses.

*AVRre trial* Aortic Valve Replacement readmission trial

^a^ Mind map were used to cue respondents’ memories before interview

^b^ Dose is number of days and calls the intervention was administered

^c^ Delivered as planned

^d^ Contextual factors, such as length of stay in local hospitals

### Study population

Patients included in the trial were initially treated and had SAVR surgery at a tertiary hospital in southeast Norway. The participants (*N* = 288) were aged > 18 years and scheduled for SAVR, singly or combined with coronary bypass surgery or/and supra coronary graft [16]. After surgery, patients in the RCT were transferred to a local hospital before discharge to their home. RCT participant inclusion and exclusion criteria are further described in the published AVRre RCT protocol [15].

### Data collection and procedure

AVRre trial participants were 1:1 block (8–12) randomized (Supplemental Fig. 1) to reduce the risk of selection and allocation bias [15]. There were two groups: the intervention group and a control group, the latter received only the usual follow-up care for SAVR surgery [15]. All data used for the process evaluation were collected prospectively during the AVRre trial, except for data obtained in the prior and post-intervention focus interviews. We used a semi-structured interview guide (Supplemental files 2 and 3) to collect information during the interviews regarding former cardiac patients’ experiences prior to the study, the nurses’ experiences during preparations for the intervention and their performance and how they perceived patient reactions to the 24/7 telephone hotline service during the intervention. We also used a mind map prior to the interview with former cardiac patients to cue and enhance their memories [20]. The mind map was constructed by the researcher and filled in by the respondent prior to the interview (a tool to frame past experiences) (Supplemental file 4). The follow-up questionnaire given 3 months after surgery comprised questions related to the use of the hotline and questions on patient-report experiences measures (PREM) from a national survey on patient experiences with discharge from hospitals; this questionnaire also had an open-ended comment field so participants could elaborate [21, 22].

The AVRre trial obtained institutional ethical approvals [15], and all participants and nurses gave written informed consent in accordance to the approval by the Regional Committees for Medical and Health Research Ethics, Health East South, Norway (approval 2013/2031–3). Trial registration number**:** ClinicalTrials.gov, NCT02522663. Consolidated Standards of Reporting Trials (CONSORT) guidelines [23] were followed in the reporting of the effectiveness of the intervention [16]. Standards for Reporting Implementation Studies (StaRI) informed the reporting of the process evaluation reported in this article [24] together with the CONSORT.

### Data analysis

Using SPSS [25], we calculated frequencies and did crosstab analyses of quantitative data from the self-report questionnaire completed 3 months after surgery. Data are presented as numbers and percentages. Fisher Exact tests were used to evaluate differences between the intervention and control groups and between other variables. Qualitative data collected prior to the AVRre trial was organized using QSR International’s NVivo 10 qualitative data analysis software [26]. Team member field notes taken during the follow-up of the AVRre trial were analyzed. Planning of the qualitative analysis approach was informed by Maxwell [27] and by Kvale and Brinkmann for the interviews [28]. Content from the TFU field notes, open-ended participant comments from the questionnaire, and the focus group were analyzed by systematic text condensation, as described by Malterud [29] and NVivo 11 Pro software [30]. All assessments and analyses were periodically discussed with one of the co-authors.

## Results

In the AVRre trial, 288 patients were included as participants. Demographic and clinical data of the study sample was reported previously [16]. Of the 127 participants in the intervention group [16], 46% used the hotline service. Ancillary analyses estimated that 81% of the readmissions in the intervention group were unavoidable (vs. 69% in the control group) [16]. These findings were relevant for the process evaluation. In the intervention group, 62.5% of the readmissions were due to a cardiac-related cause, compared to 50% for the control group (ns, *P* > 0.05; Supplemental Table 1). There was a non-significant trend toward more readmissions in the control group compared to the intervention group.

### Implementation

#### Dose

All 127 of the intervention participants received the planned structured TFUs on days 2 and 9 after discharge from the local hospitals (Supplemental Table 2). In very few cases (*N* = 4), the TFU dose deviated from the planned dose, mainly due to unexpected events. These deviations took place shortly (mainly a few hours) after unexpected events. The patients (*N* = 4) received extra TFUs because they were seriously anxious, or because follow-up was needed for a potential life-threatening complication.

#### Fidelity

Prior to conducting the AVRre trial, the content of the 24/7 manuals was analyzed, mainly using findings from the research interviews with former cardiac patients. This pre-trial assessment prompted us to make minor changes in the prioritization of the themes presented in the 24/7 hotline manual (Supplemental Table 3). A brief prospective assessment of the calls confirmed that the prioritization of the themes was quite accurate. Thus, we found that the 24/7 manual met the nurses’ expectations regarding its purpose. This is evident from the following statements made by some members of the focus group:

*“The book we had was very nice!”*

*“[This book] could actually be really useful in some GPs’ [general practitioners’] offices, as well.”*

In general, TFU nurses were satisfied overall with the manual and viewed it as a valid instrument for its purpose. These findings were supported by the prospectively collected field notes, which showed a high degree of hotline nurse compliance with the planned hotline service, facilitated by the 24/7 manual.

The pilot study did not reveal any substantial concerns regarding the design of the study, neither ones relating to logistics about the web randomization of participants nor ones relating to the TFU part of the intervention. However, the 24/7 hotline received only one call during the pilot study. Although the hotline staff nurses found the 2-h educational session to be useful, focus group analyses indicated that more pre-trial educational sessions and training would have been useful for preparing the nurses to deliver the hotline service. This was evident from some of the nurses’ comments during the focus group:

*“I would like to have been trained more before….”*

*“I don’t remember so much from that [2-hour] educational session.”*

These sentiments are consistent with the field notes, which indicated that more preparatory work would have reduced this possible barrier to delivery fidelity, especially when nurses first start working at the hotline service.

The hotline staff greatly appreciated participating in regular formal meetings, during which cases were discussed, consultations with the project coordinator and physicians we conducted, and educational sessions during the AVRre trial were done. The main conclusions derived from the field notes were that these regular interactions bolstered the nurses’ satisfaction, increased their confidence, and helped them to gain knowledge and develop skills to carry out the hotline service well. These conclusions are reflected in the TFU nurses’ statements during the focus group:

*“The education during the main trial was very good, and it was satisfying to go through the different cases.”*

*“It helped me to advance professionally. Attending the educational sessions— a huge plus.”*

*“I got more confident with time….”*

The close follow-up with the project coordinator and availability and consultations were perceived as very good and valuable:

*“It was an excellent follow-up… during the trial.”*

The field notes supported this finding that close follow-up during the hotline service increased the confidence of the nurse staff, likely facilitating the fidelity of the intervention delivery.

The SAVR participants perceived that delivery of the TFU was a valuable service. This was exemplified in the following themes derived from the content analyses: “a necessary service,” “high satisfaction,” “feeling of safety,” and “trustworthiness.” This perception of value was reflected in some comments’ participants made in the questionnaire given 3 months after their surgery:

*“I was called twice and that covered my needs.”*

*“These conversations were very important to me. It worked to calm me.”*

Moreover, the themes “reassurance” and “feeling of safety” are in line with the overall high satisfaction participants experienced, as described in the TFU field notes on the delivery of the hotline service.

### Mechanisms of impact

In the self-report questionnaire completed 3 months after surgery, the patients who actually used the hotline viewed it as very positive (58%), as a very safe way to access assistance (86%), and as a good and trustworthy post-discharge care option (91%) (Table 2). Moreover, the analysis of participants’ responses to the 24/7 hotline showed that they (1) experienced a continuum of care, (2) experienced individualized care, and (3) showed a need for accessing information in the early rehabilitation phase (Table 3). Assessment of participant responses to the 24/7 hotline, as reported by the hotline staff (focus group interviews), could be categorized into three themes: safety (reassurance and availability), high satisfaction, and a need to monitor symptoms after discharge.

**Table 2:** Patient self-report on the 24/7 Hotline in the AVRre trial

	To what degree was you satisfied with using the phone-support for information?	To what degree did you feel safety by having access to phone-support?	To what degree do you think this phone-support was a good offer?
In very large extent, N (%)	21 (38)^a^	30 (51)^e^	37 (64)^h^
Largely, N (%)	11 (20)^b^	20 (35)^f^	15 (27)^i^
To some extent, N (%)	10 (18)^c^	7 (11)^g^	3 (4)^j^
To a small degree, N (%)	1 (2)	–	1 (2)
Not at all, N (%)	1 (2)^d^	–	–
Not applicable, N (%)	13 (20)	2 (3)	2 (3)

Examples of AVRre trial participants subjective statements connected to their assessments (AVRre trial, Aortic Valve Replacement readmission trial):

^a^I have to say that the follow-up after discharge was shamefully bad, I got the best help and clearest answer from your Hotline!

^b^Thank you for saving my life. Without the Hotline, I think I would have died if you had not referred me to the hospital

^c^I think my questions should have been answered by a Cardiologist and not by surgical personnel when I used the Hotline

^d^The person I communicated with had less knowledge

^e^I called when I felt I needed to

^f^Having the opportunity to call whenever I wanted made me feel safe

^g^It was like taking Diazepam to know that I could call

^h^I hope this will be a permanent offer in the future

^i^I could just call the Hotline if I wondered about anything

^j^I am satisfied with the opportunity to use the Hotline if necessary

**Table 3:** Overview of the content analysis of AVRre trial participants’ reactions about the 24/7 telephone hotline

Patient reactions about the 24/7 telephone hotline	
Code	Patient_Hotline_Safe
Category	Patient’s experience of safety
Theme	24/7 hotline telephone service made them feel safe
**Overarching theme**	**Continuum of care**
Code	Patient_Hotline_Satisfaction
Category	Patient’s experiences with 24/7 hotline service
Theme	I was treated professionally by staff of the hotline service
**Overarching theme**	**Individualized care**
Code	Patient_Hotline_Need
Category	Post-discharge service
Theme	I want easy access to health information after hospital discharge
**Overarching theme**	**Information needed for the early rehabilitation phase**

*AVRre trial* Aortic Valve Replacement readmission trial

The analysis of the participants’ responses to the TFU revealed the following themes in the content analyses: (1) a gap in the transition of care, (2) a need for easy access to secure health information during a vulnerable phase, and (3) a desire for optimized self-care management (Supplemental Table 4). In the questionnaire given at 3 months, the participants in the intervention group who chose not to use the hotline (i.e., non-callers) had five main reasons. Ordered from most to least frequently, they chose not to because they were: highly satisfied with the TFU, in a rehabilitation facility, afraid to bother the healthcare system unnecessarily, felt safe knowing they had the opportunity to call at will, and had no pressing issues to address during the early rehabilitation phase.

### Context

Several discharging hospitals took part in the AVRre trial. In the intervention group, the percentage of readmissions for these hospitals ranged from 0 to 50%, after excluding two local hospitals that had fewer than 10 total discharges (Supplemental Table 5). These two hospitals were located in close proximity and were comparable in size and responsibilities. Despite these similarities, these two local hospitals (nos. 2 and 3) differed significantly in the total proportion of readmissions versus non-readmissions (*P* = 0.042; Fisher Exact test). These two local hospitals also showed a large difference in mean length of stay (LOS) at 4 versus 7 days.

The intervention and control groups in this cohort, overall, shared similar discharge experiences (Table 4). However, the data varied regarding their experiences with relevant discharge issues. For example, approximately 40% of the total cohort answered negatively about preparedness in case of complications or what ailments to expect after discharge. Moreover, approximately 25% of them said they were not informed about their actual medication on discharge from hospital. Furthermore, assessment of the TFU-call field notes and the patients’ written questionnaire responses 3 months after surgery showed substantial differences in the perceived quality of care between local hospitals. Patients stated:

*“All in all, very well satisfied with the result, treatment and care, and follow-up afterwards.”*

*“I am not happy with the follow-up from the local hospital.”*

**Table 4:** AVRre trial participants’ self-reports about hospital discharge experiences 3 months after surgery

	Intervention (*N* = 119) ^a^	Control ^b^ (*N* = 119)	*P*^c^
Informed about what you could do at home in the event of a relapse?			
To a very large extent, N (%)	10 (8.40)	3 (2.50)	0.835
Largely, N (%)	26 (21.8)	21 (17.6)	0.515
To some extent, N (%)	30 (25.2)	21 (17.6)	0.206
To a small extent, N (%)	19 (16.0)	33 (27.7)	0.041
Not at all, N (%)	18 (15.1)	29 (24.4)	0.103
Not applicable, N (%)	16 (13.4)	12 (10.1)	0.547
Informed about what ailments to expect after discharge?			
To a very large extent, N (%)	12 (10.1)	6 (5.10)	0.220
Largely, N (%)	30 (25.2)	29 (24.6)	1
To some extent, N (%)	34 (28.6)	37 (31.4)	0.777
To a small extent, N (%)	18 (15.1)	20 (16.9)	0.860
Not at all, N (%)	20 (16.8)	22 (18.6)	0.865
Not applicable, N (%)	5 (4.20)	4 (3.40)	1
When hospital staff evaluated my healthcare needs after discharge, did they consider what I and my relatives wanted?			
To a very large extent, N (%)	14 (11.7)	13 (10.8)	1
Largely, N (%)	40 (33.3)	28 (23.3)	0.114
To some extent, N (%)	14 (11.7)	26 (21.7)	0.056
To a small extent, N (%)	17 (14.2)	14 (11.7)	0.700
Not at all, N (%)	12 (10.0)	14 (11.7)	0.836
Not applicable, N (%)	23 (19.2)	25 (20.8)	0.872
At time of discharge, I clearly understood my responsibility for own health.			
To a very large extent, N (%)	31 (25.8)	33 (28.0)	0.884
Largely, N (%)	56 (46.7)	46 (39.0)	0.238
To some extent, N (%)	25 (20.8)	24 (20.3)	1
To a small extent, N (%)	4 (3.3)	9 (7.6)	0.253
Not at all, N (%)	2 (1.7)	4 (3.4)	0.684
Not applicable, N (%)	2 (1.7)	2 (1.7)	1
At time of discharge from hospital, I clearly understood my medication.			
To a very large extent, N (%)	44 (36.7)	45 (38.5)	1
Largely, N (%)	54 (45.0)	37 (31.6)	0.033
To some extent, N (%)	14 (11.7)	21 (17.9)	0.272
To a small extent, N (%)	2 (1.7)	10 (8.50)	0.034
Not at all, N (%)	4 (3.3)	1 (0.9)	0.370
Not applicable, N (%)	2 (1.7)	3 (2.6)	1
Were you informed about your actual medication when discharged from hospital?			
Not applicable, N (%)	2 (1.7)	2 (1.7)	1
Yes, N (%)	85 (70.8)	89 (76.1)	0.765
No, N (%)	33 (27.5)	26 (22.2)	0.561

^a^ Eight participants were unavailable to provide self-reports at the 3-month assessment, accounting for the difference in the 127 intervention participants who received the planned structured TFUs on days 2 and 9 after discharge from the local hospitals

^b^ Participants in the control group received usual care [15]

^c^ Fisher Exact test

Some patients stated that it was too far to travel to the local hospitals for cardiac rehabilitation and that they worried about the GPs’ competence to provide adequate follow-up care.

## Discussion

The AVRre trial failed to reduce 30-DACR. The high proportion of unavoidable readmissions suggested that this factor was an important part of why the intervention failed [16]. The non-significant trend toward more readmissions in the intervention group requires further study. Few clinical RCTs employing a post-discharge follow-up designed to reduce readmissions discuss why the trial failed on the primary outcome; i.e., had no effect on readmissions. It has been suggested that participation bias and lack of power to analyze sub-groups could play a role in such a negative outcome [31]. Moreover, in previous clinical RCTs that reported no or little effect, the presence of Type I errors may explain why a reduction in readmissions were not detected [32]. Another possibility offered was that the intervention itself may have inadvertently heightened participants’ awareness of early post-discharge symptoms they were experiencing, prompting them to contact the health system, and thus increase the likelihood of a readmission [32].

### Implementation

#### Evaluation of pilot study

Our process evaluation suggested some positive aspects of trial preparation and some less-than-optimal preparation for the AVRre trial. The 24/7 TFU manual was perceived as a professional tool by the hotline staff, which likely facilitated adequate delivery of the intervention. The design and content of the 24/7 manual proved to be appropriate, and demonstrated why it is important that end users participate in the developmental phase of an intervention. This early involvement ensured that the intervention content remained the same for the same group of participants. We suggest that the TFU manual had a positive impact on intervention implementation, and at least had no negative effect on the primary outcome. The manual may even have had a positive impact on improving anxiety symptoms of the post-surgical SAVR patients, because of its useful content. This content, in turn, enabled the nurses using the manual to deliver the hotline service with fidelity.

Instruction before implementation of the AVRre trial also had positive aspects and some less-than-optimal aspects. Hotline staff reported that the 2-h educational session done before the pilot study was useful. However, members of the staff said that if they had received even more instruction and training before the AVRre trial began, they might have been more confident in delivering the service right from the beginning of the intervention. Even though the SAVR participants said they were satisfied with the hotline service, more instruction and training for the hotline staff might have produced even greater participant satisfaction (greater than 58%), and perhaps better primary outcomes. Thus, these process evaluation findings of less-than-optimal intervention preparatory work can be interpreted as a barrier in reducing 30-DACR after SAVR. However, other clinical interventions in the literature reporting low delivery fidelity have produced improved outcomes [17].

Only one incoming patient telephone call was made to the hotline staff during the pilot study. A larger pilot study would have provided more pre-AVRre trial training leading to greater facility in staff handling incoming patient calls from the start. Moreover, a more extensive pilot study could have given us more information about the proportion of unavoidable readmissions to expect. This pre-trial information would have allowed us to change the primary outcome to only target the avoidable readmission rate. A larger pilot study would have also allowed a more accurate power calculation for this purpose.

#### Evaluation during the AVRre trial

The range of support available to the hotline staff was appropriate to run the hotline service, and this likely resulted in the intervention being delivered with high fidelity. The ease in learning the intervention allowed the staff to quickly gain confidence in its delivery. Our presumption was confirmed that if experienced nurses had readily available resources, they would actually deliver a high-quality support service [15]. Still, more pre-trial training might have improved the hotline service even more, leading to better primary outcomes. However, the high proportion of unavoidable readmissions in the AVRre trial cohort was likely an important contributing cause to why the intervention delivery had no impact on 30-DACR after SAVR.

The content analysis of the intervention fidelity revealed that the trial participants were mainly highly satisfied and trusted the intervention. Evidence in the literature is mixed about patient satisfaction with post-discharge telephone support [33, 34] and impact of telephone interventions on reducing readmissions [12–14, 35]. Different methods employed in the delivery of telephone support in different contexts might explain some of the heterogeneity regarding satisfaction and readmission outcomes. Therefore, it seems important to include user satisfaction and other PREMs in an evaluation of a delivered health service [36]. Our results show that the mixed methods we used in the process evaluation allowed us to obtain a richer picture of the impact of the intervention.

### Mechanism of impact

The overall high patient-reported satisfaction with the AVRre intervention, as supported by the content analyses, adds power to explaining why participants’ anxiety symptoms improved during the intervention period. While delivery of healthcare can sometimes be disjointed and episodic, participants in the AVRre trial experienced their post-discharge care as a continuum of care, which likely contributed to less anxiety. These results are consistent with the finding that providing a continuum of care after cardiac surgery leads to better health outcomes [37]. Our participants reported feeling safe, because they had easy access to secure health information, also likely contributing to less anxiety. An important finding of our process evaluation was that patients in the AVRre trial knew that they were not lost in care transition but rather experienced a continuum of care. More than 90% of the participants reported that the intervention was a good option. This result prompts questions about several aspects of post-discharge care in general.

Gaps in the continuum of care after hospital discharge are well known and reported [38]. Our process evaluation also revealed that participants experienced more individualized care and assistance in monitoring symptoms. This finding might suggest that this aspect is important for reducing symptoms of anxiety after discharge and to achieve greater satisfaction with the intervention. A more individualized approach to post-discharge hospital care has the potential to increase the quality of the care after discharge [39]. However, executing post-discharge care is complex, and interventions that are multimodal are more likely to yield lower readmissions [11, 40]. When looking at the PREM results, issues like patients’ adherence to medications and preparedness for coming home appear to be factors to target for quality improvement. Increasing the number of patient-reported outcomes after cardiac surgery is warranted [41].

### Context

Our process evaluation results showed great variability in the total proportion of readmissions between the local hospitals. Different discharge management practices/policies of local hospitals are likely to be an important driver of readmissions after SAVR; our PREM results support this suggestion. Moreover, the significant difference in total proportion of readmissions we observed between two comparable local hospitals (nos. 2 and 3) clearly demonstrates that local hospital contextual factors can influence the readmission rate both positively and negatively. The hospital in the AVRre trial with more readmissions (no. 2) also had shorter LOSs, a similar finding as that in a nationwide Norwegian study, in which shorter LOSs increased the risk of readmissions [42]. However, this finding is at variance with the literature on readmissions, which suggest that shorter LOSs do not predict readmissions [43, 44]. An important future line of research is to determine why hospitals within the same healthcare system have varying readmission rates. Uncovering this answer can provide important information that can be used to optimize discharge care after SAVR.

The quality of care provided locally was perceived as being less good to excellent by AVRre trial participants. We believe this supports our interpretation that different local hospitals’ management systems are a barrier to better discharge outcomes. Moreover, this might additionally have had moderator effects on the intervention. Participants having to travel long distances to a local hospital for follow-up seems to be a barrier to engage in local cardiac rehabilitation; this factor potentially impacted post-discharge outcomes. According to some participants’ responses, the practices and characteristics of GPs responsible for primary care can be a target for further study on how they impact the outcomes associated with the SAVR treatment. Studies of TAVI patients have similar post-discharge complications as SAVR patients [6]. Thus, our findings on readmission factors affecting optimal post-discharge care for SAVR patients might be valuable for the TAVI population as well.

### Methodological considerations

This study has multiple strengths. First, the mixed-methods approach we used allowed us to obtain a broad and deep understanding of the results. This rigorous, multilevel approach also opens up the possibility that our results can be compared in order to confirm and clarify others’ findings. Second, the intense follow-up and continuous discussions among staff during the intervention produced reliable data for carrying out a robust process evaluation. Third, using the MRC framework to guide the process evaluation and to structure the collected data was effective for helping us gain a broader understanding of the trial effects. Fourth, we used questions from a well-established PREM questionnaire, allowing comparison and contrast. Fifth, a transparent description of important intervention elements bolsters the possibility for others to conduct good replication studies and to compare results across different studies.

Our study has limitations. First, the process evaluation was not formally integrated initially into the RCT design. More prospective and tailored data collection designed to specifically conduct a process evaluation could have further enriched the findings and could have revealed ones at odds with the present findings. Second, more data on different local hospital discharge management systems could allow for adjustments of potential moderator effects on the primary outcomes. Third, due to partial retrospective data collection, we cannot rule out the possibility of recall bias. Fourth, researcher bias due to preconceptions due to prior knowledge cannot be ruled out. Fifth, lack of more contextual data limits the possibility to adjust for possible confounding effects. Sixth, if we had digital recordings of the hotline calls, this would have allowed us to conduct an even more in-depth analysis of the fidelity of the AVRre trial intervention trial delivery.

## Conclusions

We found that the intervention was well-implemented. The SAVR patients in the study were satisfied and felt safe participating in the intervention. This observation suggests that in usual care for post-discharge SAVR patients, a gap is present in the care continuum. This study adds knowledge to the importance of integrating user experiences into clinical trials as part of a process evaluation and adding knowledge for optimizing the transition of care after SAVR.

## Supplementary information

**Additional file 1: Figure S1.** CONSORT flow chart for participant selection and group assignments for the AVRre study. **Table S1.** Proportion of unavoidable readmissions in the AVRre trial and cause of 30-DACR. **Table S2.** Distribution of AVRre trial participants at local discharge hospitals. **Table S3.** Themes and headings in the 24/7 telephone manual for hotline nurses to use in the AVRre trial.** Table S4.** Overview of content analysis of SAVR patient participant reactions of the TFU in the AVRre trial. **Table S5.** Distribution of readmissions and non-readmissions in the AVRre trial and local hospitals.

**Additional file 2.** Semi-structured interview guide: Focus group -AVRre Trial.

**Additional file 3.** Semi-structured interview guide: Experiences after discharge from hospital as a cardiac surgery patient.

**Additional file 4.** Mind map used to interview former cardiac patients' in the AVRre Trial.

## Data Availability

The datasets generated and/or analyzed during the current study are not publicly available due to privacy concerns and hospital regulations but are available from the corresponding author on reasonable request.

## Abbreviations

30-DACR: 30-day all-cause readmission
AS: Aortic stenosis
AVRre: Aortic Valve Replacement Readmission trial
AVR: Aortic valve replacement
GP: General practitioner
LOS: Length of stay
MRC: Medical Research Council
ns: Not significant
PREM: Patient-report experiences measures
RCT: Randomized controlled trial
TAVI: Transcatheter aortic valve implantation
SAVR: Surgical aortic valve replacement
StaRI: Standards for Reporting Implementation Studies
TFU: Telephone follow-up
