# Supplementary material for: Facilitators of and barriers to reducing thirty-day readmissions and improving patient-reported outcomes after surgical aortic valve replacement: a process evaluation of the AVRre trial
Source: BMC Health Serv Res. 2020 Mar 27;20:256. doi: 10.1186/s12913-020-05125-5 (PMC7102432; doi:10.1186/s12913-020-05125-5)
Supplement: Supplementary file 1 — Additional file 1: Figure S1. CONSORT flow chart for participant selection and group assignments for the AVRre study. Table S1. Proportion of unavoidable readmissions in the AVRre trial and cause of 30-DACR. Table S2. Distribution of AVRre trial participants at local discharge hospitals. Table S3. Themes and headings in the 24/7 telephone manual for hotline nurses to use in the AVRre trial. Table S4. Overview of content analysis of SAVR patient participant reactions of the TFU in the AVRre trial. Table S5. Distribution of readmissions and non-readmissions in the AVRre trial and local hospitals. [file 12913_2020_5125_MOESM1_ESM.docx]

# Semi-structured interview guide: Experiences after discharge from hospital as a cardiac surgery patient

Retrospective focus group/in-depth interview guide to obtain data on what former cardiac surgery patients (i.e., not AVRre trial participants) experienced when they first got home after discharge (especially experiences in the first month).

- Do you remember how you were discharged from the hospital?
  - Anything about the discharge you would like to comment on especially?
- What do you remember from the time you first got home after hospital discharge?
  - Can I look at your mind map?
  - Can you say something about what you perceived about the care after discharge?
- Should the hospital have done anything different when you were discharged?
- What kind of impressions do you have of the rehabilitation care offers you were given?
- If you were to summarize what you experienced when you first got home after hospital discharge, what would you say?
