# Supplementary material for: Facilitators of and barriers to reducing thirty-day readmissions and improving patient-reported outcomes after surgical aortic valve replacement: a process evaluation of the AVRre trial
Source: BMC Health Serv Res. 2020 Mar 27;20:256. doi: 10.1186/s12913-020-05125-5 (PMC7102432; doi:10.1186/s12913-020-05125-5)
Supplement: Supplementary file 3 — Additional file 3. Semi-structured interview guide: Experiences after discharge from hospital as a cardiac surgery patient. [file 12913_2020_5125_MOESM3_ESM.docx]

# Semi-structured interview guide: Focus group -AVRre Trial

Retrospective focus group discussion guide to obtain data on the nurses’ experiences of the preparation and their performance on the 24/7 telephone hotline service.

- How did you experience preparations for the intervention?
  - Possible follow-up: Should we have done anything different?
- How did you experience your performance during the delivery of the intervention?
  - Possible follow-up: Was it different from that expected? If it was different, why is it different from what you expected?
- How did you experience the follow-up during the intervention?
- Was there anything that, or someone who, made it difficult for you to perform the service you were trained to do?
- What kind of impression do you have of the participants’ experiences of the hotline and follow-up telephone service?
- If you were to summarize what you were involved in, what would you say?
