# Supplementary material for: Facilitators of and barriers to reducing thirty-day readmissions and improving patient-reported outcomes after surgical aortic valve replacement: a process evaluation of the AVRre trial
Source: BMC Health Serv Res. 2020 Mar 27;20:256. doi: 10.1186/s12913-020-05125-5 (PMC7102432; doi:10.1186/s12913-020-05125-5)
Supplement: Supplementary file 4 — Additional file 4. Mind map used to interview former cardiac patients' in the AVRre Trial. [file 12913_2020_5125_MOESM4_ESM.docx]

Mind map used to interview former cardiac patients’ in the AVRre Trial

How did you experience the first month home after hospital discharge? Think about the symptoms you experienced.

Write down the key words.
